# Mechanisms of neuroplasticity linking early adversity to depression: developmental considerations

**DOI:** 10.1038/s41398-021-01639-6

**Published:** 2021-10-09

**Authors:** Tiffany C. Ho, Lucy S. King

**Affiliations:** 1 Department of Psychiatry and Behavioral Sciences and Weill Institute for Neurosciences, University of California, San Francisco, CA USA; 2 Department of Psychiatry and Behavioral Sciences, Tulane University School of Medicine, New Orleans, LA USA; 3 Department of Psychology, University of Texas at Austin, Austin, TX USA

**Keywords:** Neuroscience, Psychology, Biomarkers

## Abstract

Early exposure to psychosocial adversity is among the most potent predictors of depression. Because depression commonly emerges prior to adulthood, we must consider the fundamental principles of developmental neuroscience when examining how experiences of childhood adversity, including abuse and neglect, can lead to depression. Considering that both the environment and the brain are highly dynamic across the period spanning gestation through adolescence, the purpose of this review is to discuss and integrate stress-based models of depression that center developmental processes. We offer a general framework for understanding how psychosocial adversity in early life disrupts or calibrates the biobehavioral systems implicated in depression. Specifically, we propose that the sources and nature of the environmental input shaping the brain, and the mechanisms of neuroplasticity involved, change across development. We contend that the effects of adversity largely depend on the developmental stage of the organism. First, we summarize leading neurobiological models that focus on the effects of adversity on risk for mental disorders, including depression. In particular, we highlight models of *allostatic load, acceleration maturation, dimensions of adversity, and sensitive or critical period*s. Second, we expound on and review evidence for the formulation that distinct mechanisms of neuroplasticity are implicated depending on the timing of adverse experiences, and that inherent within certain windows of development are constraints on the sources and nature of these experiences. Finally, we consider other important facets of adverse experiences (e.g., environmental unpredictability, perceptions of one’s experiences) before discussing promising research directions for the future of the field.

## Introduction

From the earliest stages of life, environmental input interacts with the developing nervous system to influence the possible onset, maintenance, and prognosis of depression. Researchers and clinicians have long recognized that early adversity, defined here as environmental exposures that constitute deviations from the expectable environment during child development (e.g., abuse, neglect) [1, 2], contributes to risk for depression [3–6]. When individuals experience adversity, they often mobilize responses from the endocrine, immune, and nervous systems, which, in turn, engage mechanisms of neuroplasticity that prepare them to respond to learned environmental contingencies and future threats [7]. Several frameworks that link adversity and attendant stress responses to disease processes, including allostatic load [8] and accelerated maturation [9], postulate that biological weathering from chronic activation of stress response systems increases risk for mental disorder. Other models highlight the role of the timing of adversity within the context of sensitive periods of development, asking whether adversity affects experience-expectant or experience-dependent mechanisms [1, 10]. Given that depression commonly emerges during adolescence and can be detected as early as preschool age [11, 12], it is critical that we consider the fundamental principles of developmental neuroscience when elucidating the mechanisms linking early adversity to depression.

Considering that both the environment and the brain are highly dynamic across the period spanning gestation through adolescence, we propose in this review that stress-based models of depression adopt a developmental lens. Specifically, we argue that whether and how psychosocial adversity disrupts or calibrates the biological systems implicated in depression depends largely on the developmental stage of the organism, as the sources and nature of the environmental input shaping the brain—and the mechanisms of neuroplasticity involved—change across development. Although many of the concepts we describe in this review extend to mental disorders more broadly, including bipolar disorder, eating disorders, posttraumatic stress disorder (PTSD), and substance use disorders, we focus on depression as an exemplar for studying how psychosocial adversity enhances risk for negative mental health outcomes by disrupting neurodevelopment. We focus on depression because it is prevalent [13], highly comorbid with other mental disorders [14, 15], and, although linked to family history [16], appears to have limited genetic etiology compared to many other disorders [17]. Underscoring the centrality of stress to depression, many candidate biomarkers of depression, including smaller hippocampal volume [18, 19], disruptions in HPA-axis functioning [20, 21], and elevated peripheral inflammation [22, 23], are also linked with exposure to early adversity (see [24–26] for reviews).

In this review, we begin by briefly discussing the major mechanisms of neuroplasticity that unfold over the course of prenatal to adolescent development. Second, we review the leading neurobiological models of the effects of adversity on risk for mental disorder, including allostatic load, accelerated maturation, dimensional models, and sensitive period models. Third, we expound on the formulation that distinct mechanisms of neuroplasticity are implicated depending on the timing of these experiences, and that inherent within certain windows of development are constraints on the sources and nature of these experiences. Fourth, we review evidence in favor of this formulation. Finally, we discuss other important considerations regarding adverse experiences—specifically, environmental unpredictability, inescapability of exposure, and perceived severity—before summarizing emerging research themes and exciting future directions in this area.

## Mechanisms of neuroplasticity across development

**Fig. 1:**
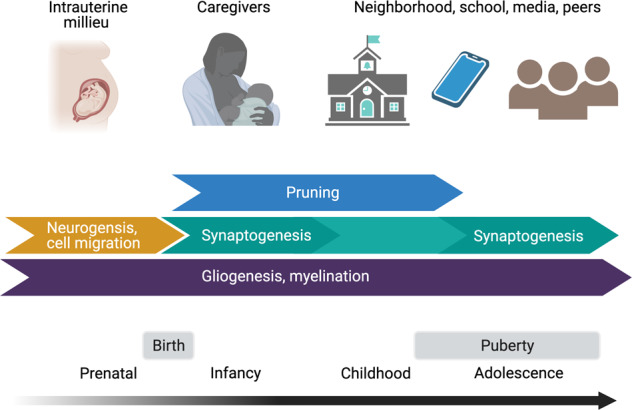
Primary sources of environmental input and mechanisms of neuroplasticity across development. Whether and how adversity disrupts or calibrates biological systems implicated in depression depends largely on the developmental stage of the organism. Primary sources of environmental input and mechanisms of neuroplasticity change across the period spanning gestation to adolescence. Maternal physiological signals transmitted to the intrauterine environment influence neurogenesis and cell migration. As children mature, dominant sources of environmental input expand, coinciding with ongoing changes in synaptic formation and remodeling, and with changes in gliogenesis and myelination. Primary sources of expected environmental input can also serve as sources of adversity that influence the neurodevelopmental processes taking place in that period. Figure created with BioRender.com.

Human brain development is a protracted process that begins in gestation and lasts for at least two decades. In Fig. 1, we illustrate the distinct mechanisms of neuroplasticity that govern the different stages of brain development from the prenatal period through adolescence. *Neurogenesis*, the formation of new brain cells, including neural stem cell/progenitor cell proliferation, neuronal migration, and neuronal differentiation, occurs primarily during embryonic development, laying the foundation for sensory processing of environmental input and postnatal experience-dependent development [27]. *Synaptogenesis*, the elaboration of synapses, is a process that unfolds rapidly across the first year of life, peaking between 2 and 4 years and again during adolescence [28–30], and supporting functional connections between regions of the brain. *Synaptic pruning*, the elimination of synapses to consolidate efficient and exploited connections, peaks between ages 2 and 10 years [29, 30]. Finally, *gliogenesis*, the formation of astrocytes, oligodendroctyes, and microglial cells, which play a role in remodeling of synapses [31, 32], and the related process of *myelination*, the increase of myelin that facilitates the structural neural connections that buttress functional connectivity, occurs throughout development at varying rates [33].

## Overview of models linking adversity to mental disorders

### Allostatic load

One prevailing framework that links stress responses systems to mental disorder centers on the concept of *allostatic load*, which is defined as the phenotypic consequences of chronic activation of stress response systems, including endocrine, immune, and neural adaptations to environmental input [34, 35]. While the physiological changes that attend exposure to adversity are helpful in the short-term by allowing the body to maintain homeostasis through changing environmental conditions, over time these initially adaptive responses produce “wear and tear” on regulatory systems. For example, *acute* exposure to threatening adversity promotes the secretion of hormones (e.g., cortisol) and inflammatory cytokines that drive changes in structural plasticity of the hippocampus and amygdala to enhance fear learning for similar events [36]. However, *chronic* stress acts through these same hormonal and immune mediators to create glutamatergic excitoxicity and atrophy in the hippocampus and amygdala linked to impaired memory and other behavioral and cognitive symptoms commonly seen in depression [37].

### Accelerated maturation

As others have noted [9, 38], the allostatic load model is limited in terms of its focus on mature systems. This framework does not address the dynamic nature of stress response systems across development—that is, responses to stress earlier in development influence future development of the organism. Theories rooted in evolutionary biology that also integrate perspectives from developmental science posit that experiences in early life tune stress response systems to features of a given environment in order to maximize fitness [9, 38–40]. Scientists have argued that for individuals raised in environments with multiple sources of threat and/or where long-term survival is uncertain, this “developmental reprioritization” often manifests as *accelerated maturation* characterized by an earlier emergence of adult-like phenotypes [9, 41].

Findings from the animal and human literature support the accelerated maturation model. For example, the transition from approach to avoidance responses to shock-paired odors that occurs across typical rat development is accelerated in rats exposed to early adversity, with an accompanying precocious amygdalar phenotype [42]. Similarly, studies in juvenile mice have shown that early adversity leads to accelerated myelination of axons in the amygdala [43]. Parallel findings have been documented in human children who experienced institutional care [44], childhood abuse [45], and who were diagnosed with PTSD [46]. Specifically, negative functional connectivity between the amygdala and prefrontal cortex during threat processing, which is thought to reflect cortical downregulation of stimulus-driven signaling in the amygdala, has been interpreted as an adult-like neural phenotype [47–49] (although see [50]) that has been associated with lower anxiety symptoms and/or better emotion regulation [47, 48] and more severe cumulative childhood adversity [51] in typically developing youth. Consistent with models of accelerated maturation, compared to typically developing children, several studies have found that children who have experienced severe maltreatment precociously exhibit this phenotype [44–46] (although see [52, 53]).

### Dimensions of adversity

Historically, theories of allostatic load and accelerated maturation did not explicitly consider the multi-dimensional nature of adversity or the potentially distinct consequences of different forms of adversity. *Dimensional models* of adversity [2, 40, 54–57], however, emphasize that it is important to elucidate core dimensions of adversity that may differentially shape brain development through distinct mechanisms. Prevailing dimensional models distinguish between adverse experiences characterized by *deprivation* (i.e., the absence of beneficial input, such as cognitive impoverishment or emotional neglect) versus those characterized by *threat* (i.e., the presence of harmful input, such as abuse). In this framework, deprivation and threat are theoretical axes of experience that elicit distinct responses in endocrine, immune, and neural systems.

Emerging findings indicate that distinguishing dimensions of adversity enhances specificity in our understanding of the environmental origins of deviations in biological development and risk for mental disorder. For example, research in humans indicates that experiences of threat, and *not* deprivation, are associated with accelerated maturation in terms of epigenetic aging, cellular age, pubertal timing, and thinning of the ventromedial prefrontal cortex [58, 59]. In addition, human children who experienced deprivation in the form of prolonged institutionalization have been found to have poorer inhibitory control and working memory [60] and difficulties in *reward-based* associative learning that contribute to higher depressive symptoms [61]. In contrast, children who were exposed to threat (e.g., living in neighborhoods with violent crime) and who were also at higher risk for developing depression, have been found to display deficits in *fear-based* learning that are associated with reductions in the volume of the amygdala and hippocampus [62]. Similar findings regarding differential effects of deprivation and threat on human brain morphometry have been documented by other groups [63–65].

It is important to note that previous research in non-human animals that linked exposure to adversity with accelerated development of amygdalar circuitry used environmental manipulations that were arguably characterized by deprivation (e.g., limited bedding, early weaning, maternal separation) [42, 43, 66]. Similarly, at least one study of human children exposed to extreme psychosocial deprivation in the form of institutionalization found evidence of accelerated maturation of amygdala-prefrontal circuitry [44]. These apparently equivocal findings may be due to the fact that deprivation can be further disaggregated into experiences involving deficits in material, cognitive, or emotional input [67]. In both humans and other species, impoverished environments characterized by material forms of deprivation (e.g., confined environments lacking visual stimulation, nutritional deficiency) are strongly linked with delays in brain growth and pubertal development [68–70]. Unlike these material forms of deprivation, deprivation of emotional input (e.g., lack of comfort from caregivers) may lead to accelerated maturation in amygdala–prefrontal circuitry because young organisms depend on their caregivers to regulate their emotional responses to everyday stressors is a species-expected experience [71, 72].

Another possible explanation for mixed findings regarding the associations of deprivation and threat with accelerated maturation is that experiences of deprivation may accelerate certain mechanisms of neuroplasticity, but not others. Supporting this formation, a recent systematic review found that deprivation, but not threat, is associated with accelerated cortical thinning of the ﻿frontoparietal, default, and visual networks [59]. Deprivation may accelerate synaptic pruning because certain sensitive periods require specific environmental input to open (discussed in more detail in the following section) [73, 74].

Whereas dimensional models of adversity focus on core aspects of adversity that underlie multiple experiences, it is also possible that specific forms of adversity, including certain types of maltreatment, have distinct effects on development. While different types of childhood maltreatment often co-occur [75, 76] and are all strongly linked with depression risk [77, 78], neuroimaging work by Teicher et al. suggest that specific types of adversity are associated with distinct regional brain patterns. For example, exposure to parental verbal abuse was associated with structural alterations in left auditory cortex and the arcuate fasciculus (a white matter tract that connects temporal and parietal regions critical for language processing), whereas witnessing domestic violence was associated with structural alterations in visual cortex and the inferior longitudinal fasciculus (a white matter tract that connects visual cortex with frontolimbic structures; see [65] for a review of these studies). In contrast, exposure to childhood sexual abuse in young women has been found to be associated with reduced gray matter volume in portions of visual cortex responsible for processing face stimuli [79] and portions of somatosensory cortex corresponding to the genital representation field [80]. If replicated in larger and diverse samples, these findings would suggest that exposures to certain *types* of early adversity have *selective* consequences on neurophenotypes. Nonetheless, given the complexity of characterizing the naturalistic environment, many studies using a specificity approach to examine the impact of maltreatment on the brain lack the requisite information to determine whether effects are related to the focal adversity or another correlated experience. As we discuss in more detail in the next section, there is also increasing evidence that the *timing* of these exposures, likely in interaction with their type, is critical for brain outcomes relevant to the pathophysiology of depression (e.g., hippocampal subfield development [81]).

### Critical or sensitive periods

*Critical or sensitive periods* are circumscribed windows of developmental plasticity during which variation in environmental input has uniquely strong and lasting effects on the brain and behavior [82]. Whereas periods of development in which environmental input has stronger effects on long-term functioning are “sensitive,” periods in which missing or extremely deficient inputs lead to the permanent loss of certain functions are “critical” [82]. Thus, critical or sensitive period models emphasize that the developmental *timing* of adversity plays a significant role in neurodevelopmental processes and risk for mental disorders [1, 10, 73]. According to these models, adverse experiences that occur outside of the perimeters of a critical or sensitive period may have a relatively limited impact on subsequent neurodevelopment. Commonly cited evidence for critical or sensitive periods in sensory and higher-order associative processing systems include amblyopia or other visual deficits resulting from visual deprivation during infancy [83], as well as difficulties in acquiring and producing language in the absence of linguistic input and symbolic expression [84].

Research studies conducted in human children who have experienced institutional care and were deprived of psychosocial input support this formulation, although it is difficult in these studies to disentangle the effects of timing from the duration of deprivation (for a review, see [85]). For instance, the English and Romanian Adoptees (ERA) study [86], a 20-year observational study of Romanian children who experienced institutionalized care and were subsequently adopted by families in the United Kingdom, and the Bucharest Early Intervention Project (BEIP) [87], a 15-year study of outcomes following a randomized control trial of high-quality foster care as an alternative to institutionalization in Romanian orphans, found that deprivation of expected input during infancy and toddlerhood in behavioral and neural outcomes. Specifically, results of the ERA indicate that, by age 6 years, children who were adopted earlier (i.e., duration of exposure to deprivation <6 months) had low levels of symptoms of behavioral disturbances; however, children who had been exposed to institutionalized care for longer durations presented with elevated symptoms of autism spectrum disorder, attention-deficit/hyperactivity disorder (ADHD), and disinhibited social engagement disorder that persisted into adulthood [88]. Further, compared to adopted adults who were not exposed to deprivation, previously institutionalized adopted adults had significantly smaller total brain volumes, which, in turn, statistically mediated the association between history of institutionalization and certain behavioral outcomes (IQ and ADHD symptoms); moreover, longer duration of institutional care was negatively associated with brain volume [89].

Within the BEIP, previously institutionalized children randomized to high quality foster care by age 31 months generally had better cognitive, behavioral, and neurodevelopmental outcomes than did children randomized to remain in “care as usual” (typically, prolonged institutionalization) [90]. Nonetheless, these children still had deficits compared to their never-institutionalized peers. For example, consistent with the data acquired during adulthood from the participants in the ERA [89], at age 8 years, both children randomized to foster care and “care as usual” children evidenced significantly smaller cortical gray matter volume compared to never-institutionalized children [91]. By implicating synaptic development in gray matter cells (i.e., dendritic branching, axonal outgrowth), these findings support sensitive period models suggesting that the lack of expected environmental input during early life affects experience-expectant mechanisms of neuroplasticity. However, children participating in the BEIP who were randomized to foster care showed recovery of cortical white matter volume and fractional anisotropy in several white matter tracts such that they were statistically indistinguishable from their never-institutionalized peers on these measures [91, 92]. Findings of recovery in relation to white matter development indicate that experience-dependent mechanisms of plasticity involving glial cells, including myelination, may explain how environmental input shapes development outside of critical or sensitive periods.

### The role of genetics in models of adversity

Although a more detailed discussion is beyond the scope of this review, children’s genotypes, and that of their parents, influence not only the development of stress response systems [93] and brain phenotypes [94, 95], but also the types of environments they encounter and how these environments affect their outcomes [96–99]. Beyond the diathesis stress framework, which posits that poor health emerges when genetic vulnerability meets with environmental adversity, well-known evolutionary models, including *biological sensitivity to context* and *differential susceptibility theory*, posit that certain genotypes may confer either elevated risk or enhanced wellbeing depending on whether environmental conditions are negative or positive [100]. While it has become clear that the effects of individual genetic variants on behavior are too small to be reliability detected [101], burgeoning genome-wide association studies are now informing the development of polygenic scores—aggregate indices of genetic influences—that can be used to more directly examine the role of genotype in children’s experiences of adversity and their outcomes following these experiences [102]. In addition to the presence of certain genes affecting responses to adversity, experiences of adversity may influence genetic expression through epigenetic mechanisms, such as DNA methylation, histone modifications, and non-coding RNA (for reviews, see [103, 104]). In fact, many candidate gene and epigenome-wide association studies have documented correlations between early adversity and alterations in the epigenome [105–108]. In turn, alterations that change the expression of genes in the brain affect various molecular processes, including mechanisms of neurodevelopment, that affect risk for depression (for a review, see [104]). Nonetheless, the extent to which variations in the epigenome (e.g., DNA methylation) are a causal mechanism linking adversity to mental disorder, or are simply a biomarker of vulnerability, remains unclear and is an active area of research [109].

### Integrating and extending existing models

#### Environmental influences generate distinct effects on brain circuitry due to developmental constraints in sources of input and in mechanisms of plasticity

Integrating knowledge gained through existing models linking early adversity to mental disorders, we propose that there are developmental constraints both on mechanisms of neuroplasticity and the sources environmental input (see Fig. 1). In addition, interactions between dynamic neurodevelopmental processes and changing sources of environmental input implicate distinct mechanisms of neuroplasticity corresponding to the developmental timing of adversity, with likely distinct phenotypic consequences across all levels of analysis from cells to behavior. Thus, the current framework bridges sensitive period and dimensional models by emphasizing the importance of both timing and type of adversity, while recognizing how the concept of allostatic load critically explains the mediating endocrine, immune, and neural regulators of early adversity on risk for mental disorders, including depression.

For example, we posit that experiences of deprivation during the later postnatal stages of development (e.g., childhood, adolescence) have a lesser impact on brain and behavioral outcomes compared to earlier stages of development (e.g., infancy), as experience-expectant mechanisms of plasticity are no longer engaged. Infancy and toddlerhood are studded with critical or sensitive periods (e.g., for language and attachment) in which missing environmental input may lead to the permanent loss of specific functions supported by specific groups of neurons and synaptic connections. In contrast, later childhood is more likely to involve experience-dependent learning supported by gliogenesis and myelination. Infancy presents limited opportunity for environmental input beyond the immediate caregiving environment such that infants are especially vulnerable to exposure to and the consequences of deprivation in the form of neglect [110–113]. In contrast, although infants and young children are exposed to abuse by their caregivers [113], sources of adverse experiences involving threat may increase with development as the range of environmental input expands (although high individual variability in patterns of adversity across the life course is expected). Specifically, as children mature, they become capable of engaging with their neighborhood, schools, peers, and social and non-social media independently. Although expanding environmental input provides new opportunities for enrichment, it may also entail direct exposure to hostile school environments, peer conflict, and community violence.

Below, we illustratively review human and non-human animal literature investigating the consequences of adversity during the prenatal, infant, childhood, and adolescent periods on mechanisms of neuroplasticity and risk for depression. Based on the current framework, we focus on the consequences of experiences of deprivation in infancy and emphasize the effects of threat-related adversity during later childhood and adolescence. We center experience-expectant mechanism as the primary processes relevant in infancy and experience-dependent mechanisms as the primary processes by which adversity influences phenotypic outcomes later in development.

## Sources of environmental input interact with neuroplasticity across development

### Prenatal period

A growing body of literature documents the effects of psychosocial stress or adversity (as well as biochemical insults that are beyond the scope of this review) experienced by mothers both during pregnancy and prior to conception on increased risk for mental disorders in their offspring [114]. As detailed in Fig. 1, the primary source of environmental input during fetal development is in the intrauterine milieu. Thus, although the fetus is not directly exposed to adversity, variation in the intrauterine environment associated with maternal stress or adversity may influence prenatal brain development through mechanisms of plasticity (e.g., neurogenesis, synaptogenesis) that lay the foundation for subsequent neurobiological development and future risk for depression and other mental disorders. As such, the prenatal period represents a critical period of development. Experimental evidence from research conducted in rodents demonstrates that inducing prenatal adversity leads to anhedonic behaviors in offspring that are analogous to human depression (e.g., withdrawal from social play, reduced sucrose preference) [115]. In humans, numerous studies indicate that women’s experiences of adversity during pregnancy are associated with risk for mental disorders, including depression, in their child and adult offspring [116–119].

The association between prenatal psychosocial adversity and offspring risk for mental disorder is hypothesized to be mediated, at least in part, by the signals the fetus receives in utero [120, 121]. As in other life periods, stress response systems, including the hypothalamic pituitary adrenal (HPA) axis and the immune system, are activated in response to environmental threat during pregnancy. Ensuing changes in maternal physiology, including increases in levels of glucocorticoids and elevations in inflammation, may influence fetal neurodevelopment by affecting placental functioning and/or by passing through the placenta and the fetal blood–brain barrier [122–124]. Although these maternal physiological signals may help prepare the fetus for the postnatal environment [125], they may also have adverse consequences, particularly when the postnatal environment does not match prenatal “expectations” [126]. For example, women’s exposure to stressful life events during pregnancy is linked to differences in the structural and functional connectivity of amygdala–prefrontal circuitry in newborn infants [127]. Higher maternal prenatal levels of interleukin-6, a pro-inflammatory cytokine that is upregulated in response to psychosocial stress, are associated with lower fractional anisotropy in amygdala–prefrontal white matter tracts (uncinate fasciculus) in newborn infants and poorer subsequent cognitive functioning at age 12 months [128]. Similarly, elevated maternal cortisol during pregnancy was associated with stronger functional connectivity of the amygdala in newborn female infants and higher subsequent internalizing symptoms at age 24 months [129]. Finally, maternal prenatal stress and adversity may also affect epigenetic processes that alter neurodevelopment [103]. For example, in rodents, offspring of mothers exposed to chronic unpredictable stress (e.g., aversive sounds, confinement, food deprivation) during pregnancy had decreased hippocampal histone acetylation [130]. In humans, women’s perceived stress during the second trimester was associated with mildly increased methylation of glucocorticoid-related genes in their placental tissue, which was, in turn, associated with reduced fetal coupling of heart rate and movement [131]. Together, these studies point to endocrine, inflammatory, and epigenetic mechanisms by which prenatal psychosocial experiences of the mother engender an adverse intrauterine environment for the fetus that affects their subsequent development in ways that may increase their risk for negative health outcomes.

Importantly, in addition to fetal programming by maternal adversity during pregnancy, maternal adversity that occurred prior to conception may influence offspring brain development by affecting epigenetic processes, the quality of the intrauterine environment, and the child’s postnatal experiences [132, 133]. For example, women’s exposure to childhood maltreatment has been linked with lower cortical gray matter volume in their newborn infants [134]. Clearly, distinguishing the effects of maternal adversity prior to conception and during pregnancy on offspring neurodevelopment and risk for depression is challenging given that adversity is likely to be correlated across life stages. To help address this challenge, King et al. [135] have recently developed a novel measure to assist in the simultaneous assessment of parent and child adversity, including adversity occurring during discrete stages of the parents’ life corresponding to the child’s development (i.e., prior to conception, prenatally, postnatally).

### Infancy

Although rapid brain development occurs in gestation, postnatal experiences nevertheless potentiate experience-expectant mechanisms of plasticity throughout the first year of life. During infancy, caregivers are the primary source of environmental input, providing for instrumental needs (e.g., food and shelter) and delivering experience-expectant psychosocial stimulation (e.g., cognitive stimulation and nurturance) [67]. Infancy is a window of opportunity for the positive effects of environmental enrichment from caregivers on long-term development. However, given that caregivers are the primary source of environmental input in this period, they can also be the primary source of adversity. Specifically, caregivers may either fail to deliver experience-expectant input—as is the case in neglect—or create harmful input—as is the case in abuse. Perhaps because they spend the majority of their time with their caregivers during what is often a challenging time for parents [136], infants under age one year are more likely to be exposed maltreatment than are children in any other developmental period [110].

The prevalence of adversity in the form of maltreatment during infancy is especially concerning because this period involves cascading critical or sensitive periods, including for the development of language and the formation of attachment bonds that undergird socioemotional competency [1]. Thus, violations of the expectable environment during infancy may be particularly detrimental, leading to atypical circuit development with potentially irreversible consequences for associated behavior. Specifically, because infancy involves rapid synaptogenesis followed by pruning of unused connections, deprivation during this period may lead to infants failing to form important connections and experiencing severe synapse elimination [74]. Non-human animal models of early deprivation and enrichment support this formulation [137, 138]. In humans, institutionalized children who are deprived of individualized input from caregivers often fail to develop organized—let alone secure—attachment styles and are more likely to develop disorders of social relatedness that persist across childhood [88, 139, 140]. They also evidence lasting differences in brain function detectable as lower EEG power in the alpha frequency band and reduced total brain and gray matter volumes [89, 91, 141]. Although depression is not assessed in infancy, the attachment system organizes infants’ regulation of emotion, and early insecure and disorganized attachment is associated with internalizing symptoms across childhood, including depressive symptoms [142].

Abused infants may develop structural and functional differences in amygdala–hippocampal–prefrontal circuits underlying fear learning that may have long-term consequences for psychiatric risk despite the fact that these experiences are not explicitly remembered [143]. Human infant neuroimaging, including MRI, is burgeoning [144]; however, few studies have investigated the neural correlates of variation in the environment during the postnatal period (i.e., most studies focus on the neonatal brain and therefore speak to the impact of *prenatal* experiences, although see [145, 146]). Moreover, ethical considerations place obvious limits on allowable paradigms for investigating fear learning behavior in human infants. Thus, most of what we know about the impact of postnatal adversity during infancy on fear learning behavior and circuitry comes from studies of non-human animals experimentally exposed to stressors. Exposure to threat in infancy—either directly in the form of footshock or through limited bedding and nesting paradigms that elicit maternal abuse—appears to enhance sensitivity to subsequent fear learning that is mediated by the amygdala. Specifically, rat pups exposed to threat in the form of multiple shocks at postnatal day 17 showed enhanced subsequent contextual fear learning in response to a tone paired with a single footshock (for days, weeks, and even months thereafter) [147]. Exposure to abuse in infancy also appears to disrupt behavior and amygdala activity in response to attachment cues. For example, compared to rat pups raised in typical conditions, those raised in conditions that lead to maternal abuse showed reduced preference for their mother’s odor, decreased time nursing, and enhanced engagement of the amygdala in response to the mother’s odor [148].

As previously discussed, deprivation of nurturance may be unique compared to other forms of deprivation in its impact on stress response systems. Although it is perhaps more intuitive that exposure to threat affects fear-related circuitry, based on cross-species evidence that non-threatening input from caregivers regulates stress responses during infancy [149], deprivation of nurturance from a caregiver in this period may also converge on the same amygdala–hippocampal–prefrontal circuits. Animal research strongly supports this formulation. In young rat pups, maternal presence reduces shock-induced corticosterone release and its subsequent influence on amygdala activity, thereby blocking amygdala-mediated aversion learning [150]. Compared to rat pups reared in standard environments who have amnesia for fear learning in infancy, those who were deprived of maternal interaction subsequently demonstrated lasting retention of learned associations between conditioned stimuli and threats [151]. Thus, infants who are deprived of important caregiver interactions are likely to experience differences in the foundational development of fear circuits and aversion or fear learning behaviors that place them at greater risk for the development of depression.

### Childhood

As infants develop and enter their preschool and school-age years, they gain greater independence from their caregivers and encounter a wider range of environmental input. Although input from caregivers remains essential, particularly for stress regulation and emotion learning [71, 152], school entry involves critical additional sources of environmental input. Interactions with teachers, peers, and media in school involve new sources of enrichment, but can also entail exposure to new forms of adversity such as bullying, and, unfortunately in the U.S., threats of violence in the form of school shootings and preparation for these incidents as early as Kindergarten [153].

Compared to infants, young and school-age children have greater capacity for cognitive processing of their experiences and may form long-term memories of adversity. Thus, from the perspective of life history strategy, the effects of earlier and ongoing adverse experiences on children’s representations of the self and the environment may become more evident. In the context of threat, these models may involve guilt, shame, and expectations that the environment is unpredictable or dangerous [154]. In fact, depression can be diagnosed as early as the preschool years [12] and is marked by the complex self-conscious emotion of guilt [155]. Exposure to adversity partially mediates the association between family history of depression and preschool depression [156], which predicts depression through later childhood and adolescence [157].

Stress paradigms based on exposure to chronic threat (e.g., daily footshock or immobility restraint stress) in non-human juvenile animals have chronicled have identified differences in amygdala-dependent avoidance learning and appetitive behaviors as well as hippocampal- and prefrontal-dependent memory and attention deficits following adversity [158–160]. These non-human animal responses resemble symptoms of human depression. Non-human animals exposed to chronic threat also showed sustained growth of dendritic spines in the amygdala that tracked with the development of generalized fear responses [161], but significant atrophy in the hippocampus and prefrontal cortex that coincided with behavioral changes consistent with anhedonia [161, 162].

Compared to non-human animal paradigms, studies of human children raised in threatening environments involve more unpredictable adversity. Moreover, a wider range of behavioral and cognitive outcomes have been examined in humans, providing a more nuanced understanding of the effects of early adversity on risk for depression (and mental disorders more generally). For instance, children exposed to threatening experiences showed diminished effortful control and cognitive reappraisal [163], they also showed enhancements in certain aspects of cognition, such as cognitive flexibility [164], and attentional biases to negative stimuli [165] that—consistent with accelerated maturation models—may be associated with more mature amygdala–prefrontal connectivity [166]. During childhood, threatening environmental input acts on experience-dependent mechanisms of plasticity involved in synaptic pruning and myelination, likely affecting neurobiological development in ways that reflect adaptations to the child’s specific environment. Overall, these results indicate that the detection and encoding of negative stimuli that support survival in threatening environments but that potentially contribute to the onset and maintenance of depression may be preferentially conserved and strengthened among children consistently exposed to adversity.

### Adolescence

With the initiation of puberty, adolescence represents a second period of synaptogenesis and synaptic pruning, with experience-dependent remodeling of brain circuits underlying complex social behaviors [167, 168]. Although the “activating” and “organizing” effects of adrenal and gonadal hormones on brain plasticity are beyond the scope of the current review (see [169–171] for recent reviews on this topic), increases in adrenal and gonadal hormones across pubertal development have been shown to be associated with changes in gray matter volume and white matter connections across human adolescence [172–174], with a growing literature demonstrating associations between these brain regions and their connections with behavioral responses to socially relevant stimuli [168].

From an evolutionary perspective, by the time of adolescence, children have acquired the input necessary from their caregivers for early survival and have established the skills that only the caregiving environment can provide (e.g., regulation of emotion through the attachment system) [170]. Therefore, they begin to explore outside of the immediate caregiving environment to gain new input that is important for long-term survival (e.g., establishing independence, broadening affiliative networks, fostering social integration). Peers, especially, become a critical source of environment input and drive exploratory and approach behaviors. Whereas positive peer relationships can buffer adolescents’ affective and hormonal stress responses [175], there is also evidence that when in the presence of peers versus not, adolescents show increased activation in regions involved in social cognition following social exclusion [176], and diminished cognitive control to positive social cues when anticipating rewards, concurrent with greater activation in the orbitofrontal cortex [177]. Further, when in the presence of peers versus parents, adolescents demonstrated weaker recruitment between the ventral striatum and regions involved in social cognition, self-awareness, and in representations of the self and others (insula, temporoparietal juncture) [178]—a profile that may reflect reduced top-down input on reward sensitivity. Female adolescents exposed to hostile school environments evidence greater prefrontal (specifically rostral anterior cingulate) responses to social exclusion, which mediate the association between exposure to hostile school environments and behavioral problems [179]. Thus, enhanced relevance of peers during adolescence may have both protective and stress-enhancing effects.

Mounting evidence also indicate that puberty opens a window of neuroendocrine plasticity, with implications for how adolescents mobilize stress responses systems. Specifically, according to the “pubertal stress recalibration” hypothesis [180], neuroendocrine plasticity in puberty permits experiences of more recent positive input to remediate the effects of earlier experiences of adversity. For example, there is evidence that patterns of stress-evoked cortisol reactivity and regulation following extreme forms of adversity change during puberty to match current environmental conditions. Among children exposed to adversity in the form of institutionalization in infancy who were subsequently raised in supportive adoptive families, a recent study found that within-individual increases in pubertal stage corresponded to increases in cortisol reactivity to a laboratory-based stressor [180]. It is currently unclear what are the long-term consequences of HPA-axis recalibration for risk for depression; nonetheless, these studies suggest pubertal normalization of the HPA-axis among children exposed to early adversity but later enrichment. Unlike adopted children who experience dramatic changes in their environments, however, children who are exposed to early adversity and who continue to be raised in their families of origin are likely to remain in adverse environments through adolescence [181]. For these children, puberty may lead to changes in HPA-axis functioning that are not necessarily health-enhancing [182, 183]. It will be important in future research to examine whether pubertal recalibration is also instantiated in brain measures reflecting mechanisms of neuroplasticity.

## Other considerations

Although an exhaustive survey of all features of adversity is beyond the scope of this review, we wish to briefly highlight additional aspects of adversity that are important to consider when interpreting the studies reviewed. These aspects may be especially relevant when seeking to translate findings from non-human animal models of stress to risk for depression following adversity in humans.

First, environmental unpredictability (i.e., stochastic variation in environmental conditions that increase risk for death and disease) and other related concepts from evolutionary biology are thought to play a unique role in how adversity shapes the development of traits that are predictive of life history behaviors (e.g., mating) and mental wellbeing (e.g., relationship satisfaction) [40, 184]. Although operationalizing environmental unpredictability is complex, it is important to determine in future research whether there are sensitive periods of development for responding to environmental unpredictability, what are the consequences of encountering environmental unpredictability early in development and how these coincide with other dimensions of adversity (e.g., accelerated or delayed maturation and deprivation or threat), and, finally, what are the underlying mechanisms of plasticity that mediate stress responses to environmental unpredictability across development [185].

Second, the inescapability of a stressor, which has been robustly tested in “learned helplessness” paradigms where the controllability and predictability of stressors (e.g., aversive stimuli like electric shocks or immobilization restraint) are varied, has a rich history in non-human animal models of depression [186]. Even single exposures to inescapable stressors have been shown to induce greater changes in the brain (dendritic spine outgrowth in the amygdala) and behavior (fear generalization) than exposure to chronic unpredictable stressors [187]. The psychological construct of perceived entrapment in humans has been strongly linked with depression and even suicide risk [188]. The extent to which perceived controllability or entrapment may moderate the effects of adversity on brain and behavioral systems across development remains an active area of research.

Third, compared to accidents and other non-intentional negative life events, interpersonal adversities often involving family or peers (e.g., abuse, bullying, domestic violence) are strongly associated with depression [189, 190] and comorbid disorders [191], perhaps due to their often chronic and generative nature and their unique effects on an individual’s sense of self-worth and trust in others [192–194]. Some studies have identified distinct effects of childhood interpersonal adversity on the developing brain (for example [195]). Interpersonal adversity, however, is extremely difficult to model in non-human animals. Mice are often the model organism for examining adversity models of depression but are prey species that may lack the capacity for complex interpersonal representations. Although chronic defeat paradigms are akin to physical threats of interpersonal victimization in humans, these models do not capture more insidious forms (e.g., ongoing harassment, conflict, or emotional maltreatment) of adversity that impact higher-order emotions which have a central role in depression (e.g., shame, guilt).

Finally, it is critical for health care providers to monitor occurrences of childhood maltreatment and evaluate and treat any harm, physical or otherwise, regardless of the child’s perceptions of these experiences; however, individuals’ perceptions of the severity of adverse experiences and daily hassles also play a critical role in triggering depressive episodes and may partly explain the link between early adversity and subsequent depression and other disorders [196–198]. Recent work from our group has shown that perceived stress mediated the association between the objective severity of childhood adversity and depressive symptoms in adolescents during the early months of the COVID-19 pandemic [199]. Further, we have found that, above and beyond the objective severity of adverse experiences in childhood, the perceived severity of these experiences was associated with anxiety symptoms in adolescents and fractional anisotropy of the uncinate fasciculus [200, 201]. As with interpersonal adversity, this aspect of experience is extremely challenging to model in non-human animals. Further, questions regarding the role of perceptions of adverse experience intersect with an understanding of when children develop meta-cognitive abilities that help them evaluate and interpret past experiences. It is possible that as these abilities develop, behavioral responses to earlier experiences change or emerge.

## Future directions

Over the last 50 years, our understanding of psychosocial adversity and the capacity of the brain to adapt to one’s changing environment has evolved, deepening our knowledge of the mechanisms through which experiences such as abuse and neglect influence biobehavioral functioning. These advances have had a direct impact on the study of depression and other mental disorders that are fundamentally characterized by pathological responses to stress. In this review, we have summarized and reconciled many of the influential models in the field by emphasizing the importance of considering both type and timing of adverse experiences. We have highlighted that changes in environmental inputs across development coincide with changes in mechanisms of neuroplasticity, implicating different sources of adversity and neurodevelopmental processes based on developmental stage. Given the complexity of this research and the varied challenges in operationalizing early adversity across species, as well as measuring its effects on neural and behavioral phenotypes, many open questions remain. Below, we highlight but a few of the research trends that continue to gain momentum.

Understanding the long-term behavioral consequences, including risk for depression (as well as other related disorders, including ADHD, bipolar disorder, eating disorders, PTSD, and substance use disorders), of prematurely terminated or shortened windows of developmental plasticity following chronic or severe exposure to adversity remains an active area of research. One speculation is that precocious termination of sensitive periods—particularly following threatening experiences—can protect the organism from the possibility that negative inputs monopolize the developing brain [9]. However, such premature closing of windows of plasticity constrains opportunity to learning; enriching inputs are unable to refine neural circuits in order to scaffold subsequent development [202]. Despite difficulty in mapping sensitive periods across mammalian species, many of the neurobiological mechanisms that generate sensitive periods appear to be common [82, 203, 204]. We require continued work on the molecular mechanisms that regulate the initiation and termination of sensitive periods [204], and the extent to which specific types of adversities are more relevant for explaining these processes.

With respect to elucidating timing effects of adversity on depression-related outcomes, a critically understudied developmental period has been pregnancy. Women’s hormonal and inflammatory milieus undergo dramatic changes across pregnancy [123, 205], suggesting that the impact of maternal adversity on fetal brain development may vary across gestation. Information about how the timing of prenatal adversity moderates its impact on the intrauterine milieu and fetal neurodevelopment has important implications for prevention and intervention. Although the question of prenatal timing has been explored in humans [206, 207], the field has yet to reach definitive conclusions about whether and how the timing of prenatal adversity influences offspring outcomes [122]. Ongoing research efforts, including the NIH-funded Environmental influences on Child Health Outcomes study [208, 209], which leverages 69 longitudinal birth cohorts in the United States in order to investigate how early adversity and environmental exposures that span behavioral, biological, chemical, physical, and social domains affect neurodevelopmental outcomes in over 50,000 children, will be critical in clarifying the potentially enduring effects of prenatal adversity across multiple stages of development (i.e., from infancy to adulthood).

Another important area of research with significant treatment implications is disentangling dose of adversity from timing. In humans, earlier exposure to adversity is almost always confounded with dose effects (i.e., earlier adversity is correlated with later adversity; “the earlier the better” findings from interventions are confounded by the fact that children who receive the intervention later may have remained in adverse conditions longer). Recent results from suggest that there are dose-response associations between length of institutionalization and total brain volume and regional gray matter surface area in frontocingulate regions [89, 91]. As reviewed above, however, children randomized to high-quality foster care in place of institutionalization appear to recover in terms of measures of white matter [91, 92]. These findings therefore align more strongly with the perspective of a sensitive period model, as the mechanisms of brain plasticity involving neuronal development are engaged early in development whereas experience-dependent mechanisms of plasticity involving white matter development remain active throughout childhood and adolescence. Thus, there may be a specific set of neurophenotypes (e.g., functional connectivity, gray matter volumes, cortical thinning, measures of myelin content) that are differentially sensitive to the effects of early adversity depending on the developmental stage and nature of that adversity. To our knowledge, no studies have sought to systematically test this formulation.

From the perspective of facilitating translational psychiatry, we require cross-species investigative efforts to gain a more complete picture of how early adversity leads to changes in genetic, epigenetic, cellular, molecular, and micro- and macro-scale brain circuit phenotypes that contribute to a heightened risk for depression. While there are appreciable limitations of non-human animal models to explain human conditions and clear restrictions on the type of environmental manipulations that can be applied in humans, there are nonetheless opportunities for both forward (e.g., identifying specific molecular regulators of plasticity) and backward (e.g., inspiring new environmental assays or examining specific points in development that were previously ignored) translation. For instance, epidemiological data demonstrate that, in terms of sex assigned at birth, depression affects female more than male individuals, particularly during the adolescent period when depression often emerges [11, 210]. However, most non-human animal work characterizes the effects of adversity on the brain and associated behavioral phenotypes of depression in male animals. Many of the classic stress responses to threat (e.g., fleeing) are unique to male rodents; moreover, there is clear evidence that early adversity leads to sex-specific consequences in dendritic morphometry in rodents [211, 212]. Developing standardized behavioral assays explaining sex differences in depression vulnerability—and the heterogeneous symptoms of depression more generally—is currently an active area of research that stands to be informed by results in human studies [213, 214].

## Conclusions

Early adversity is one of the strongest predictors of depression, and poorer mental health more generally, throughout the lifespan [3, 4, 6] in part due to the effects of adverse experiences on neurodevelopment [2, 10, 54]. In this review, we have surveyed several existing models of how adversity shapes the brain circuitry underlying risk for depression. Bridging and expanding on these models, we proposed a framework for understanding how early adversity increases risk for depression and other mental disorders that is centered on principles of neurodevelopment. In addition to emphasizing that the mechanisms of neuroplasticity change across the period spanning gestation to adolescence, we highlighted developmental changes in the sources and nature of the environmental input that influences the brain. From the perspective of this framework, we discussed existing human and non-human animal data, while acknowledging other theoretical stances and aspects of adversity that scientists must consider and further reconcile with extant models. Finally, we summarized ongoing areas of research that are poised to enhance our understanding of how early adversity contributes to risk depression. As leaders in the field recently noted, “no single conceptual model likely accounts for the entire range of complex effects of adversity on neurodevelopment” [73]. Nevertheless, by integrating cross-species research that considers the core dimensions that underlie adverse experiences as well as the timing of these experiences in the context of development, we move closer to a comprehensive account of these complex effects. Given the unfortunate prevalence of experiences such as abuse and neglect and the role of childhood maltreatment in the global burden of mental disorders [77, 215], we anticipate that understanding the mechanisms by which early adversity affects neurodevelopment represents one of the greatest opportunities for translational psychiatry to uncover fundamental insight into the etiology and treatment of depression.
